# Quality of life of women who underwent breast cancer treatment relative to sociodemographic, behavioral, and clinical factors

**DOI:** 10.31744/einstein_journal/2024AO0585

**Published:** 2024-03-14

**Authors:** Angélica Atala Lombelo Campos, Maria Teresa Bustamante-Teixeira, Rafaela Russi Ervilha, Vivian Assis Fayer, Jane Rocha Duarte Cintra, Renata Mendes de Freitas, Daniela Pereira de Almeida, Maximiliano Ribeiro Guerra

**Affiliations:** 1 Universidade Federal de Juiz de Fora Juiz de Fora MG Brazil Universidade Federal de Juiz de Fora, Juiz de Fora, MG, Brazil.; 2 Programa de Pós Graduação em Saúde Coletiva Universidade Federal de Juiz de Fora Juiz de Fora MG Brazil Programa de Pós Graduação em Saúde Coletiva, Universidade Federal de Juiz de Fora, Juiz de Fora, MG, Brazil.; 3 Instituto Oncológico Hospital Nove de Julho Juiz de Fora MG Brazil Instituto Oncológico, Hospital Nove de Julho, Juiz de Fora, MG, Brazil.

**Keywords:** Breast neoplasms, Quality of life, Sociodemographic factors, Life style, Activities of daily living, Surveys and questionnaires

## Abstract

**Objective:**

Patients with cancer often undergo multiple extended treatments that decrease their quality of life. However, the quality of life of women with breast cancer after they undergo treatment remains underexplored in Brazil. Therefore, this study determined sociodemographic, behavioral, and clinical factors related to the post-treatment quality of life of women with breast cancer.

**Methods:**

This cross-sectional study involved 101 women diagnosed with breast cancer between 2014 and 2016 and treated at a Brazilian Oncology Reference Service. Data were collected from them using face-to-face surveys. Quality of life was evaluated using the European Organization for the Research and Treatment of Cancer Core Quality of Life questionnaire (EORTC QLQ-C30) and EORTC Breast Cancer-specific Quality of Life questionnaire (EORTC QLQ-BR23). The data collected were analyzed using Student’s t-test and Mann-Whitney U test.

**Results:**

The median score on the global health, functional, and symptom scales of the EORTC QLQ-C30 was 75.00 (Interquartile range=33.33), 75.99 (Standard deviation [SD]=19.26), and 19.67 (SD=16.91), respectively. The mean score on the functional and symptom scales of the EORTC QLQ-BR23 was 61.89 (SD=17.21) and 20.12 (SD=16.94), respectively. Furthermore, higher post-treatment quality of life was found to be associated with being aged 50 or more, being Black, having eight or more years of education, having a partner, having a paying job, receiving treatment from the private healthcare system, having a higher income, living in the municipality where healthcare services are availed, engaging in physical activity, not smoking, being more religious, having more social support, not being overweight, having no comorbidities, and undergoing lumpectomy.

**Conclusion:**

Sociodemographic, behavioral, and clinical factors significantly impact the quality of life of women who undergo breast cancer treatment. Implementing interventions that improve health and reducing inequalities in the access to healthcare services can improve the quality of life of these patients.

## In Brief

Higher post-treatment quality of life of women with breast cancer is linked to being aged 50 or more, being Black, having 8 or more years of education, having a partner, having a paying job, receiving care from private healthcare, having a high per capita income, residing in the municipality where the service is availed, engaging in physical activity, not smoking, greater religiosity, having more social support, having a normal weight, having no comorbidities, and undergoing lumpectomy.

## Highlights

Sociodemographic, clinical, and lifestyle factors impact the quality of life of breast cancer survivors.

Breast cancer therapy may affect future perspectives and emotional, cognitive, and sexual function.

Some aspects of quality of life still require attention from health professionals.

**Figure:**
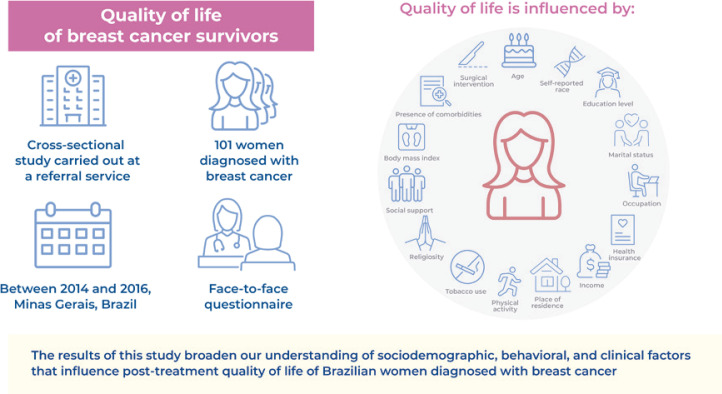

## INTRODUCTION

In the last decade, the survival rate of women with breast cancer has improved globally, mainly due to advancements in screening programs and therapies.^(1-3)^ Undergoing medical treatment is crucial for preserving life. However, it can also lead to social and psychological transformations and cause lasting damage to one’s life.^(4-6)^ One should decide whether to undergo treatment based on the treatment’s potential benefits and consequences and whether the benefits outweigh the consequences.^(4,7,8)^

Patients with cancer often undergo multiple extended treatments that cause pain and fatigue and disrupt functional ability, social connectedness, sense of well-being, and quality of life (QOL).^(7)^ In healthcare, QOL is assessed to gauge the impact of the disease and treatment on the patient’s emotional, social, physical, and overall well-being, as well as to guide clinical decisions.^(4,6,8)^

Many studies have examined QOL in the context of breast cancer. However, the QOL of women with breast cancer after they undergo treatment remains underexplored in Brazil.

## OBJECTIVE

Therefore, this study aimed to determine sociodemographic, behavioral, and clinical factors related to the quality of life of women with breast cancer after they undergo chemotherapy, radiotherapy, or both.

## METHODS

### Study design, participants, and the sample

This cross-sectional study was conducted at a referral center for public and private oncology care in Minas Gerais, Brazil. It was approved by the Ethics Committee of *Universidade Federal de Juiz de Fora* (CAAE: 05341019.5.0000.5147; # 3.749.693). The target population was women diagnosed with breast cancer between 2014 and 2016 residing in the state of Minas Gerais and receiving follow-up care at an oncology center. Based on analytic cases in the Cancer Hospital Registry, we identified 230 such women. Women undergoing chemotherapy, radiotherapy, or palliative therapy at the time of data collection were excluded.

For 230 eligible women, an estimated prevalence of QOL disorders of 50%, an error of 8%, and a confidence interval of 95%, a minimum of 92 participants was found to be a suitable sample size. Considering a 20% loss, the sample to be recruited was increased to 111.

We attempted to recruit all eligible women, but 57 of them could not be contacted by telephone despite at least three attempts, 45 declined to participate, and 27 did not attend their scheduled recruitment sessions despite confirming appointments on three separate occasions. Therefore, the final sample comprised 101 of the eligible women.

### Data collection

Data were collected in two stages. The first stage of data collection was performed between 2018 and 2019. In this stage, we collected information about the participants’ diagnosis, treatment, staging, and tumor profiling from their medical records. The second stage of data collection was carried out in 2019. In this stage, the participants were called for a face-to-face survey to address identification issues and collect data on QOL, sociodemographic factors, behavioral factors, and clinical factors.

### Variables

The dependent variable QOL was measured using the European Organization for the Research and Treatment of Cancer Core Quality of Life questionnaire (EORTC QLQ-C30) and EORTC Breast Cancer-specific Quality of Life questionnaire (EORTC QLQ-BR23).^(9-11)^ With 30 questions, the EORTC QLQ-C30 is a general questionnaire that evaluates symptoms that occurred in the previous two weeks.^(11)^ It has a functional, symptom, and global health scale. The functional scale has subscales for physical, role, emotional, cognitive, and social functions. The symptom scale has subscales for fatigue, nausea, pain, dyspnea, insomnia, loss of appetite, constipation, diarrhea, and financial difficulties.

The EORTC QLQ-BR23 is designed for breast cancer and consists of 23 questions.^(11)^ It has a functional and symptom scale. The functional scale has subscales for body image, future perspectives, sexual functioning, and sexual satisfaction. The symptom scale has subscales for the side effects of systemic therapy, concerns about hair loss, arm-related symptoms, and breast-related symptoms.

The independent variables were sociodemographic factors, behavioral factors, and clinical factors. The sociodemographic factors were age (<50 years/ ≥50 years), self-reported race (white/black), education level *(i.e*., number of years of education) (<8 years/ ≥8 years), marital status (live with a partner/do not live with a partner), occupation (*i.e*., whether engaged in a paying job) (yes/no), type of healthcare (public/private), per capita income (≥half of the minimum wage/ <half of the minimum wage), and place of residence (municipality where the service is availed/other municipality). The behavioral factors were self-reported eating habits (good or very good/fair, poor, or very poor), level of physical activity (active or inactive/sedentary), tobacco use (ex-smoker or never smoked/smoker), alcohol consumption (≥4 drinks on one occasion/<4 drinks on one occasion), religiosity (≥8 points/ <8 points) and social support (≥45 points/ <45 points). The clinical factors were surgical intervention (lumpectomy/mastectomy with or without reconstruction), body mass index (normal weight/overweight), presence of comorbidities (*i.e*., at least one other concomitant disease) (yes/no), and stage (initial - 0, I, or II/advanced - III).

Social support was assessed using the Social Support Questionnaire – Short Form, which comprises six questions. For each question, respondents indicate the number of people available to provide support and the degree of satisfaction with the support received.^(12,13)^ Resultingly, we obtained a numerical index and a satisfaction index. We summed the scores of the numerical and satisfaction indices.

Religiosity was assessed using the Duke University Religiosity Index, which consists of five items measuring three dimensions of religious involvement related to health outcomes.^(14,15)^ We summed the scores of the three dimensions.

The level of physical activity was measured using the International Physical Activity Questionnaire – Short Form (IPAQ). This questionnaire evaluates the activities one performed (frequency, intensity, and duration) in the last week.^(16)^ Respondents who did not engage in physical activity for at least ten consecutive minutes were considered sedentary. Those who engaged in physical activity for less than 150 minutes were considered inactive. Those who engaged in physical activity for more than 150 minutes were considered active.

### Data analysis

We first calculated the absolute and relative frequencies of each variable. To evaluate the correlation between the scores on the EORTC QLQ-C30 and those on the EORTC QLQ-BR23, we used Spearman’s rank correlation coefficients. Next, we evaluated the normality of continuous scales using the Kolmogorov-Smirnov test to determine the appropriate statistical test. We employed Student’s *t*-test for variables with a normal distribution and Mann–Whitney U test for those with a non-parametric distribution. All statistical analyses were performed using STATA^®^ (StataCorp LLC) software, assuming a significance level of 5% for statistical inference.

## RESULTS

Among the 101 participants, most were over 50 years of age (70.3%), identified themselves as white (59.4%), had more than eight years of education (59.4%), lived with a partner (53.5%), did not have a paying job (64.0%), received healthcare from the public healthcare system (55.4%), had a per capita income equal to or more than half of the minimum wage (86.6%), and lived in the same municipality as the hospital (80.2%). Regarding behavioral factors, most respondents reported having good eating habits (67.3%), were not sedentary (76.2%), were ex-smokers or had never smoked (91.1%), had less than 4 drinks on one occasion (83.2%), scored less than average on the religiosity scale (64.4%), and scored less than average on the social support questionnaire (52.6%). Regarding clinical factors, most respondents were overweight (64.9%), had comorbidities (66.3%), underwent lumpectomy surgery (91.0%), and were diagnosed in the early stages of the disease (78.2%).

Table 1 presents respondents’ scores on the EORTC QLQ-C30 and EORTC QLQ-BR30. The median score on the global health scale of the EORTC QLQ-C30 was 75.00 (Interquartile Range [IQR]=33.33). The mean score on the functional and symptom scales was 75.99 (Standard Deviation [SD]=19.26) and 19.67 (SD=16.91), respectively. In the EORTC QLQ-BR23, the mean score on the functional and symptom scales was 61.89 (SD=17.21) and 20.12 (SD=16.94), respectively.

**Table 1:** Respondents’ scores on the EORTC QLQ-C30 and EORTC QLQ-BR23

	Mean	Standard deviation	Median	Interquartile range	p value of Kolmogorov-Smirnov test
EORTC QLQ-C30					
Global health scale*	74.26	19.62	75.00	33.33	0.009
Functional scale*	75.99	19.26	80.00	24.44	0.118
Physical function	81.19	18.27	86.67	20.00	0.002
Role function	78.71	28.49	100	33.33	<0.001
Cognitive function	65.43	31.45	75.00	50.00	<0.001
Emotional function	67.16	31.04	83.33	50.00	0.003
Social function	90.26	21.51	100	0.00	<0.001
Symptom scale**	19.67	16.91	15.38	23.08	0.096
Fatigue	28.05	28.22	22.22	44.44	0.001
Nausea	6.44	14.52	0.00	0.00	<0.001
Pain	32.51	34.27	16.67	66.67	<0.001
Dyspnea	5.61	14.19	0.00	0.00	<0.001
Insomnia	30.03	38.44	0.00	66.67	<0.001
Loss of appetite	9.90	26.05	0.00	0.00	<0.001
Constipation	24.75	37.31	0.00	33.33	<0.001
Diarrhea	5.94	17.89	0.00	0.00	<0.001
Financial difficulties	17.82	31.82	0.00	33.33	<0.001
EORTC QLQ-BR23					
Functional scale*	61.89	17.21	66.67	19.05	0.053
Body image	82.59	24.83	91.67	25.00	<0.001
Future perspectives	50.49	42.85	66.67	100	<0.001
Sexual functioning	30.03	30.55	33.33	50.00	<0.001
Sexual satisfaction	52.54	38.76	66.67	100	0.036
Symptom scale**	20.12	16.94	16.67	21.90	0.100
Side effects of systemic therapy	18.6	17.72	14.29	23.81	0.003
Concerns about hair @@loss	35.35	44.05	0.00	100	0.001
Arm-related symptoms	26.18	28.99	11.11	44.44	<0.001
Breast-related symptoms	17.41	20.35	8.33	25.00	<0.001

* Higher scores on these scales indicate better quality of life; ** Higher scores on these scales indicate worse quality of life.

Concerning the subscales of the functional scale of the EORTC QLQ-C30, the median score for physical, role, social, cognitive, and emotional function was 86.67, 100, 100, 75.00, and 83.33, respectively. In the symptom scale, the median score was 22.22 for fatigue, 0.00 for nausea, dyspnea, insomnia, loss of appetite, constipation, diarrhea, and financial difficulties, and 16.67 for pain.

Regarding the subscales of the functional scale on the EORTC QLQ-BR23, the median score was 91.67 for body image, 66.67 for future perspectives and sexual satisfaction, and 33.33 for sexual functioning. In the symptom scale, the median score was 14.29, 0.00, 11.11, and 8.33 for side effects of systemic therapy scale, concerns about hair loss, arm-related symptoms, and breast-related symptoms, respectively.

Table 2 presents the results of analyzing the correlation between scores on the EORTC QLQ-C30 and those on the EORTC QLQ-BR23. The global health scale of the EORTC QLQ-C30 was positively correlated with the body image, future perspectives, and sexual satisfaction scales of the EORTC QLQ-BR23 and negatively correlated with the side effects of systemic therapy, concerns about hair loss, arm-related symptoms, and breast-related symptoms scales of the EORTC QLQ-BR23.

**Table 2:** Spearman correlation coefficients between the scale values of the QLQ-C30 and QLQ-BR23 questionnaires

	Functional scale	Body image	Future perspectives	Sexual functioning	Sexual satisfaction	Symptom scale	Side effects of systemic therapy	Concerns about hair loss	Arm-related symptoms	Breast-related symptoms
Global health scale	0.295^**^	0.265^**^	0.251^*^	0.114	0.312^*^	-0.515^**^	-0.480^**^	-0.347^*^	-0.394^**^	-0.329^**^
Functional scale	0.544^**^	0.359^**^	0.260^**^	0.367^**^	0.305^*^	-0.678^**^	-0.642^**^	-0.412^*^	-0.650^**^	-0.320^**^
Physical function	0.352^**^	0.195	0.098	0.297^**^	0.338^**^	-0.562^**^	-0.509^**^	-0.262	-0.507^**^	-0.290^**^
Role function	0.373^**^	0.247^*^	0.152	0.225^*^	0.193	-0.527^**^	-0.502^**^	-0.301	-0.518^**^	-0.201^*^
Emotional function	0.469^**^	0.405^**^	0.317^**^	0.204^*^	0.057	-0.554^**^	-0.507^**^	-0.522^**^	-0.572^**^	-0.275^**^
Cognitive function	0.405^**^	0.254^*^	0.188	0.276^**^	0.246	-0.522^**^	-0.537^**^	-0.202	-0.412^**^	-0.333^**^
Social function	0.252^*^	0.128	0.138	0.282^**^	0.130	-0.267^**^	-0.317^**^	-0.350^*^	-0.253^*^	-0.006
Symptom scale	-0.480^**^	-0.277^**^	-0.271^**^	-0.262^**^	-0.339^**^	0.707^**^	0.626^**^	0.419^*^	0.585^**^	0.459^**^
Fatigue	-0.482^**^	-0.291^**^	-0.222^*^	-0.267^**^	-0.380^**^	0.551^**^	0.544^**^	0.411^*^	0.433^**^	0.317^**^
Nausea	-0.014	-0.084	-0.137	0.074	-0.117	0.287^**^	0.284^**^	0.136	0.228^*^	0.224^*^
Pain	-0.296^**^	-0.196	-0.163	-0.138	-0.094	0.580^**^	0.370^**^	0.506^**^	0.601^**^	0.386^**^
Dyspnea	-0.001	0.072	-0.097	0.009	-0.197	0.252^*^	0.263^**^	0.082	0.178	0.185
Insomnia	-0.223^*^	-0.023	-0.043	-0.291^**^	-0.261^*^	0.370^**^	0.472^**^	-0.034	0.257^**^	0.217^*^
Loss of appetite	-0.202^*^	-0.163	-0.098	-0.228^*^	0.088	0.322^**^	0.389^**^	0.192	0.178	0.198^*^
Constipation	-0.326^**^	-0.274^**^	-0.187	-0.054	-0.164	0.295^**^	0.198^*^	0.137	0.267^**^	0.271^**^
Diarrhea	-0.110	-0.157	-0.045	0.049	-0.114	0.227*	0.302**	0.044	0.105	0.167
Financial difficulties	-0.431**	-0.320**	-0.259**	-0.129	-0.155	0.468**	0.349**	0.409*	0.499**	0.250^*^

* Statistical significance of p<0.05; ** Statistical significance of p<0.01.

The functional scale of the EORTC QLQ-C30 and its subscales were negatively correlated with the side effects of systemic therapy, concerns about hair loss, arm-related symptoms, and breast-related symptoms scales of the EORTC QLQ-BR23 and positively correlated with the body image, future perspectives, sexual functioning, and sexual satisfaction scales of the EORTC QLQ-BR23.

The symptom scale of the EORTC QLQ-C30 and its subscales were negatively correlated with the body image, future perspectives, sexual functioning, and sexual satisfaction scales of the EORTC QLQ-BR23 and positively correlated with the side effects of systemic therapy, concerns about hair loss, arm-related symptoms, and breast-related symptoms scales of the EORTC QLQ-BR23.

Table 3 presents the results of determining differences in respondents’ scores on the EORTC QLQ-C30 and EORTC QLQ-BR23 based on sociodemographic factors. Women aged 50 or more (*versus* younger women) scored better on the emotional function scale of the EORTC QLQ-C30 and the body image, future perspectives, and breast-related symptoms scales of the EORTC QLQ-BR23. However, they had worse scores on the sexual functioning scale of the EORTC QLQ-BR23.

**Table 3:** Quality of life of the women who participated in the study on the scales of the QLQ-C30 and QLQ-BR23 questionnaires according to sociodemographic characteristics

		Age			Self-reported race			Education level			Marital status			Occupation			Type of healthcare			Per capita income			Place of residence		
							
		<50 years	≥50 years	p value	White	Black	p value	≥8 years	<8 years	p value	Live with a partner	Do not live with a partner	p value	Yes	No	p value	Private	Public	p value	≥Half of the minimum wage	<Half of the minimum wage	p value	Municipality where the service is availed	Other municipality	p value
EORTC QLQ-C30, n (%)α		30 (29.7)	71 (70.3)		60 (59.4)	41 (40.6)		60 (59.4)	41 (40.6)		54 (53.5)	47 (46.5)		36 (36.0)	64 (64.0)		45 (44.6)	56 (55.4)		84 (86.6)	13 (13.4)		81 (80.2)	20 (19.8)	
Global health scale*β	M (SD)	70.0 (22.6)	76.1 (18.1)		75.6 (19.6)	72.4 (19.8)		74.4 (20.9)	73.9 (17.8)		77.3 (20.2)	70.4 (18.5)		77.3 (21.4)	72.8 (18.6)		77.0 (19.5)	72.0 (19.6)		76.8 (18.6)	57.7 (17.5)		76.5 (18.7)	65.0 (20.9)	
	Me (IR)	70.8 (41.7)	83.3 (25.0)	0.197	79.2 (33.3)	66.7 (25.0)	0.423	83.3 (33.3)	75.0 (25.0)	0.726	83.3 (41.7)	66.7 (25.0)	0.074	83.3 (29.2)	66.7 (29.2)	0.159	83.3 (25.0)	70.8 (33.3)	0.173	83.3 (25.0)	50.0 (8.3)	0.001	83.3 (33.3)	66.7 (33.3)	0.033
Functional scale*^Ω^	M (SD)	71.7 (21.3)	77.8 (19.2)		75.1 (20.4)	77.3 (17.6)		78.8 (18.8)	71.8 (19.4)		80.3 (18.1)	71.1 (19.5)		79.2 (16.9)	74.1 (20.5)		78.9 (18.4)	73.7 (19.8)		79.1 (16.5)	55.0 (22.7)		76.8 (19.1)	72.6 (20.0)	
	Me (IR)	75.6 (29.9)	80.0 (20.0)	0.146	76.7 (26.7)	82.2 (24.4)	0.564	84.4 (22.2)	75.6 (26.7)	0.071	86.7 (24.4)	75.6 (31.1)	0.016	82.2 (27.8)	78.9 (28.9)	0.204	84.4 (22.2)	77.8 (27.8)	0.181	81.1 (20.0)	62.2 (31.1)	<0.001	80.0 (22.2)	74.4 (25.6)	0.375
Physical functionβ	M (SD)	81.1 (16.8)	81.2 (18.9)		79.7 (19.3)	83.4 (16.5)		83.9 (17.6)	77.2 (18.7)		84.9 (16.6)	76.9 (19.2)		85.2 (14.9)	78.8 (19.8)		82.9 (19.4)	79.8 (17.4)		82.8 (18.2)	69.2 (15.3)		82.1 (17.9)	77.7 (19.6)	
	Me (IR)	86.7 (26.7)	86.7 (20.0)	0.769	86.7 (26.7)	86.7 (26.7)	0.372	86.7 (20.0)	80.0 (26.7)	0.036	86.7 (20.0)	80.0 (33.3)	0.024	90.0 (20.0)	80.0 (26.7)	0.129	86.7 (20.0)	83.3 (26.7)	0.152	86.7 (20.0)	66.7 (20.0)	0.003	86.7 (13.3)	73.3 (40.0)	0.506
Roles functionβ	M (SD)	72.8 (33.8)	81.2 (25.8)		77.5 (31.9)	80.5 (22.8)		83.3 (27.8)	71.9 (28.5)		82.7 (28.9)	74.1 (27.5)		81.5 (24.2)	77.1 (30.9)		84.8 (26.8)	73.8 (29.1)		82.1 (24.8)	56.4 (38.2)		80.9 (26.9)	70.0 (33.6)	
	Me (IR)	83.3 (50.0)	100 (33.3)	0.298	100 (33.3)	83.3 (33.3)	0.848	100 (33.3)	66.7 (33.3)	0.009	100 (33.3)	66.7 (33.3)	0.034	100 (33.3)	100 (33.3)	0.737	100 (16.7)	83.3 (33.3)	0.018	100 (33.3)	66.7 (50.0)	0.008	100 (33.3)	75.0 (50.0)	0.174
Emotional functionβ	M (SD)	52.5 (33.3)	70.9 (29.2)		64.9 (31.7)	66.3 (31.4)		69.2 (30.5)	59.9 (32.5)		69.6 (30.5)	60.6 (32.2)		65.9 (28.6)	64.9 (33.4)		68.7 (28.5)	62.8 (33.7)		70.0 (28.1)	33.9 (31.3)		66.0 (32.2)	62.9 (28.7)	
	Me (IR)	62.5 (58.3)	75.0 (41.7)	0.006	75.0 (50.0)	75.0 (50.0)	0.834	75.0 (41.7)	75.0 (50.0)	0.114	75.0 (41.7)	66.7 (58.3)	0.131	75.0 (45.8)	75.0 (50.0)	0.806	75.0 (50.0)	75.0 (58.3)	0.459	75.0 (41.7)	25.0 (41.7)	0.001	75.0 (50.0)	70.8 (37.5)	0.434
Cognitive functionβ	M (SD)	67.2 (30.8)	67.1 (31.4)		67.5 (31.2)	66.7 (31.2)		69.7 (30.3)	63.4 (32.1)		74.7 (29.9)	58.5 (30.3)		71.3 (29.9)	64.3 (31.5)		71.5 (29.4)	63.7 (32.1)		69.8 (29.9)	50.0 (36.4)		67.1 (31.2)	67.5 (31.3)	
	Me (IR)	75.0 (33.3)	83.3 (50.0)	0.921	83.3 (41.7)	66.7 (50.0)	0.916	83.3 (41.7)	66.7 (66.7)	0.362	83.3 (33.3)	66.7 (50.0)	0.003	83.3 (50.0)	66.7 (58.3)	0.268	83.3 (16.7)	66.7 (66.7)	0.248	83.3 (50.0)	66.7 (50.0)	0.052	83.3 (50.0)	66.7 (50.0)	0.969
Social functionβ	M (SD)	90.0 (21.3)	90.4 (21.8)		89.2 (23.3)	91.9 (18.7)		90.3 (20.6)	90.2 (22.9)		93.2 (17.9)	86.9 (24.8)		96.3 (12.7)	86.9 (24.7)		90.4 (21.5)	90.2 (21.7)		94.6 (13.7)	65.4 (38.2)		91.1 (20.9)	86.7 (23.9)	
	Me (IR)	100 (16.7)	100 (0.0)	0.542	100 (8.3)	100 (0.0)	0.816	100 (16.7)	100 (0.0)	0.671	100 (0.0)	100 (16.7)	0.115	100 (0.0)	100 (16.7)	0.022	100 (0.0)	100 (8.3)	0.953	100 (0.0)	66.7 (50.0)	0.001	100 (0.0)	100 (16.7)	0.117
Symptom scale**^Ω^	M (SD)	21.8 (20.2)	18.8 (15.4)		21.1 (18.9)	17.6 (13.4)		19.2 (17.3)	20.4 (16.5)		16.7 (16.4)	23.1 (16.9)		15.7 (14.9)	21.6 (17.7)		19.7 (18.1)	19.7 (16.0)		17.5 (14.8)	31.7 (20.1)		19.6 (16.8)	20.0 (17.9)	
	Me (IR)	15.4 (20.5)	15.4 (25.6)	0.416	15.4 (26.9)	15.4 (15.4)	0.274	17.9 (25.6)	15.4 (20.5)	0.728	12.8 (17.9)	20.5 (23.1)	0.059	12.8 (16.7)	19.2 (24.4)	0.096	17.9 (25.6)	15.4 (20.5)	0.993	12.8 (23.1)	25.6 (17.9)	0.003	15.4 (25.6)	19.2 (19.2)	0.924
Fatigueβ	M (SD)	33.3 (31.1)	25.8 (26.8)		31.8 (30.6)	22.5 (23.5)		28.1 (29.2)	27.9 (27.1)		24.1 (25.8)	32.6 (30.4)		24.0 (26.5)	29.9 (29.1)		27.6 (28.5)	28.4 (28.3)		24.3 (25.7)	50.4 (30.9)		27.9 (28.0)	28.3 (29.8)	
	Me (IR)	22.2 (44.4)	11.1 (44.4)	0.209	22.2 (50.0)	11.1 (33.3)	0.168	22.2 (44.4)	11.1 (33.3)	0.776	16.7 (33.3)	22.2 (44.4)	0.149	16.7 (38.9)	22.2 (55.6)	0.415	22.2 (44.4)	11.1 (33.3)	0.744	11.1 (38.9)	55.6 (33.3)	0.004	22.2 (44.4)	11.1 (50.0)	0.993
Nauseaβ	M (SD)	12.2 (20.5)	3.9 (10.3)		6.7 (17.1)	60.1 (9.7)		7.2 (16.9)	5.3 (10.2)		5.5 (14.1)	7.4 (15.1)		5.6 (11.3)	6.2 (15.3)		6.3 (16.0)	6.5 (13.4)		4.8 (12.0)	15.4 (24.0)		5.5 (13.4)	10.0 (18.3)	
	Me (IR)	0.0 (16.7)	0.0 (0.0)	0.055	0.0 (0.0)	0.0 (16.7)	0.157	0.0 (0.0)	0.0 (16.7)	0.627	0.0 (0.0)	0.0 (16.7)	0.306	0.0 (8.3)	0.0 (0.0)	0.676	0.0 (0.0)	0.0 (8.3)	0.585	0.0 (0.0)	0.0 (16.7)	0.073	0.0 (0.0)	0.0 (16.7)	0.157
Painβ	M (SD)	29.4 (29.3)	33.8 (36.3)		32.5 (36.5)	32.5 (31.2)		30.6 (34.2)	35.4 (34.6)		28.7 (32.6)	36.9 (35.9)		25.0 (32.5)	36.7 (35.0)		32.2 (36.5)	32.7 (32.7)		30.7 (34.8)	46.1 (29.8)		32.3 (34.3)	33.3 (35.0)	
	Me (IR)	16.7 (50.0)	16.7 (66.7)	0.866	16.7 (66.7)	16.7 (50.0)	0.599	16.7 (50.0)	16.7 (50.0)	0.330	16.7 (50.0)	16.7 (66.7)	0.208	16.7 (41.7)	33.3 (66.7)	0.084	16.7 (66.7)	16.7 (58.3)	0.681	16.7 (58.3)	50.0 (33.3)	0.072	16.7 (66.7)	16.7 (58.3)	0.958
Dyspneaβ	M (SD)	7.8 (16.8)	4.7 (12.9)		5.6 (15.2)	5.7 (12.7)		6.7 (16.0)	4.1 (11.0)		4.9 (13.6)	6.4 (14.9)		2.8 (9.3)	6.8 (15.9)		6.7 (16.8)	4.8 (11.8)		4.8 (12.8)	7.7 (19.9)		4.9 (13.0)	8.3 (18.3)	
	Me (IR)	0.0 (0.0)	0.0 (0.0)	0.338	0.0 (0.0)	0.0 (0.0)	0.662	0.0 (0.0)	0.0 (0.0)	0.501	0.0 (0.0)	0.0 (0.0)	0.573	0.0 (0.0)	0.0 (0.0)	0.210	0.0 (0.0)	0.0 (0.0)	0.790	0.0 (0.0)	0.0 (0.0)	0.761	0.0 (0.0)	0.0 (0.0)	0.443
Insomniaβ	M (SD)	17.8 (29.9)	35.2 (40.6)		32.8 (40.0)	26.0 (36.1)		23.9 (34.7)	39.0 (42.1)		24.1 (36.9)	36.8 (36.9)		23.1 (33.6)	33.8 (40.9)		31.1 (37.2)	29.2 (39.7)		28.9 (38.3)	35.9 (35.6)		32.1 (39.6)	21.7 (32.9)	
	Me (IR)	0.0 (33.3)	33.3 (66.7)	0.051	0.0 (66.7)	0.0 (33.3)	0.414	0.0 (33.3)	33.3 (66.7)	0.078	0.0 (33.3)	33.3 (66.7)	0.049	0.0 (33.3)	0.0 (66.7)	0.229	33.3 (66.7)	0.0 (66.7)	0.565	0.0 (66.7)	33.3 (66.7)	0.514	0.0 (66.7)	0.0 (50.0)	0.269
Loss of appetiteβ	M (SD)	13.3 (27.1)	8.5 (25.6)		8.3 (23.5)	12.2 (29.6)		8.3 (25.0)	12.2 (276)		4.3 (17.2)	16.3 (32.5)		7.4 (21.2)	11.5 (28.6)		9.6 (27.2)	10.1 (25.4)		7.9 (24.1)	17.9 (29.2)		9.5 (26.5)	11.7 (24.8)	
	Me (IR)	0.0 (0.0)	0.0 (0.0)	0.154	0.0 (0.0)	0.0 (0.0)	0.568	0.0 (0.0)	0.0 (0.0)	0.306	0.0 (0.0)	0.0 (0.0)	0.022	0.0 (0.0)	0.0 (0.0)	0.729	0.0 (0.0)	0.0 (0.0)	0.744	0.0 (0.0)	0.0 (33.3)	0.083	0.0 (0.0)	0.0 (0.0)	0.517
Constipationβ	M (SD)	26.7 (33.2)	23.9 (39.1)		27.8 (38.4)	20.3 (35.6)		25.6 (37.0)	23.6 (38.2)		24.7 (35.6)	24.8 (39.6)		23.1 (34.6)	25.0 (38.9)		27.0 (37.8)	22.6 (37.1)		25.4 (37.9)	12.8 (21.7)		25.5 (38.1)	21.7 (34.7)	
	Me (IR)	0.0 (66.7)	0.0 (33.3)	0.378	0.0 (50.0)	0.0 (33.3)	0.243	0.0 (50.0)	0.0 (33.3)	0.723	0.0 (33.3)	0.0 (33.3)	0.800	0.0 (33.3)	0.0 (33.3)	0.896	0.0 (33.3)	0.0 (33.3)	0.382	0.0 (33.3)	0.0 (33.3)	0.425	0.0 (33.3)	0.0 (33.3)	0.766
Diarrheaβ	M (SD)	10.0 (24.9)	4.2 (13.7)		6.1 (18.9)	5.7 (16.5)		7.2 (18.5)	4.1 (16.9)		6.8 (18.7)	4.9 (16.9)		5.5 (14.9)	6.2 (19.6)		6.7 (16.8)	5.4 (18.8)		3.9 (13.1)	20.5 (34.8)		4.9 (16.8)	10.0 (21.9)	
	Me (IR)	0.0 (0.0)	0.0 (0.0)	0.296	0.0 (0.0)	0.0 (0.0)	0.951	0.0 (0.0)	0.0 (0.0)	0.247	0.0 (0.0)	0.0 (0.0)	0.680	0.0 (0.0)	0.0 (0.0)	0.731	0.0 (0.0)	0.0 (0.0)	0.349	0.0 (0.0)	0.0 (33.3)	0.021	0.0 (0.0)	0.0 (0.0)	0.208
Financial difficultiesβ	M (SD)	24.4 (15.0)	15.0 (30.2)		20.6 (35.3)	13.8 (25.8)		17.8 (32.2)	17.9 (31.7)		12.3 (28.4)	24.1 (34.5)		9.3 (24.7)	22.4 (34.7)		14.1 (30.6)	20.8 (32.8)		13.1 (28.8)	43.6 (34.4)		18.5 (32.9)	15.0 (27.5)	
	Me (IR)	0.0 (33.3)	0.0 (0.0)	0.077	0.0 (33.3)	0.0 (33.3)	0.652	0.0 (33.3)	0.0 (33.3)	0.979	0.0 (0.0)	0.0 (33.3)	0.031	0.0 (0.0)	0.0 (33.3)	0.037	0.0 (0.0)	0.0 (33.3)	0.168	0.0 (0.0)	33.3 (33.3)	0.001	0.0 (33.3)	0.0 (33.3)	0.886
EORTC QLQ BR-23, n (%)α																									
Functional scale*^Ω^	M (SD)	59.1 (20.6)	63.1 (15.6)		60.8 (16.0)	63.5 (18.9)		61.9 (18.8)	61.9 (14.7)		64.3 (17.6)	59.1 (16.5)		62.5 (20.6)	61.5 (15.3)		62.8 (16.9)	61.2 (17.6)		62.1 (17.5)	58.4 (14.4)		61.4 (17.4)	63.7 (16.6)	
	Me (IR)	64.6 (32.1)	66.7 (17.3)	0.353	62.5 (21.1)	70.8 (16.7)	0.428	66.7 (23.8)	62.5 (19.1)	0.999	66.7 (21.2)	61.9 (19.0)	0.135	66.7 (27.1)	62.5 (19.0)	0.773	66.7 (20.8)	66.7 (21.4)	0.646	66.7 (19.0)	58.3 (19.0)	0.474	66.7 (19.0)	69.0 (23.8)	0.598
Body imageβ	M (SD)	74.2 (26.2)	85.9 (23.6)		82.8 (24.4)	82.3 (25.8)		81.2 (27.6)	84.5 (20.2)		81.9 (26.4)	83.3 (23.2)		77.1 (29.3)	85.7 (21.8)		84.8 (21.6)	80.8 (27.2)		82.8 (25.9)	78.2 (18.8)		82.8 (26.2)	81.7 (18.6)	
	Me (IR)	83.3 (25.0)	100 (25.0)	0.001	91.7 (25.0)	91.7 (25.0)	0.994	91.7 (25.0)	91.7 (25.0)	0.896	91.7 (25.0)	91.7 (25.0)	0.864	91.7 (33.3)	91.7 (25.0)	0.146	91.7 (25.0)	91.7 (25.0)	0.665	91.7 (25.0)	75.0 (16.7)	0.104	91.7 (25.0)	87.5 (20.8)	0.168
Future perspectivesβ	M (SD)	33.3 (42.9)	57.7 (41.0)		43.3 (42.2)	60.9 (42.1)		44.4 (41.5)	59.3 (43.8)		43.8 (42.4)	58.2 (42.5)		49.1 (44.7)	51.6 (42.4)		42.2 (38.5)	57.1 (45.3)		52.8 (42.1)	35.9 (44.0)		50.6 (43.2)	50.0 (42.6)	
	Me (IR)	0.00 (66.7)	66.7 (100)	0.009	33.3 (100)	66.7 (66.7)	0.037	33.3 (100)	66.7 (100)	0.083	33.3 (100)	66.7 (100)	0.092	50.0 (100)	66.7 (100)	0.798	33.3 (66.7)	66.7 (100)	0.078	66.7 (100)	0.0 (66.7)	0.175	66.7 (100)	66.7 (100)	0.893
Sexual functioningβ	M (SD)	43.9 (31.4)	21.2 (28.4)		30.3 (29.5)	29.7 (32.4)		35.8 (31.6)	21.5 (21.2)		43.5 (28.5)	14.5 (25.2)		41.2 (30.2)	23.2 (28.9)		32.6 (30.3)	27.9 (30.8)		29.6 (29.3)	32.1 (35.7)		28.4 (28.6)	36.7 (37.7)	
	Me (IR)	50.0 (33.3)	16.7 (50.0)	0.004	33.3 (50.0)	16.7 (50.0)	0.793	33.3 (66.7)	0.0 (33.3)	0.020	33.3 (33.3)	0.0 (16.7)	<0.001	33.3 (41.7)	8.3 (50.0)	0.003	33.3 (50.0)	16.7 (50.0)	0.388	33.3 (50.0)	33.3 (50.0)	0.961	33.3 (50.0)	33.3 (75.0)	0.476
Sexual satisfactionβ	M (SD)	54.5 (37.9)	51.3 (66.7)		49.2 (38.5)	59.6 (39.4)		54.2 (38.3)	49.1 (40.6)		55.3 (36.6)	44.4 (44.8)		62.9 (36.2)	44.1 (39.8)		57.1 (38.3)	48.4 ( 39.3)		50.7 (39.4)	57.1 (37.1)		50.0 (38.9)	63.6 (37.9)	
	Me (IR)	50.0 (66.7)	66.7 (100)	0.752	50.0 (83.3)	66.7 (66.7)	0.326	66.7 (66.7)	66.7 (100)	0.645	66.7 (66.7)	66.7 (100)	0.368	66.7 (66.7)	33.3 (66.7)	0.069	66.7 (66.7)	33.3 (100)	0.392	66.7 (100)	66.7 (66.7)	0.688	66.7 (83.3)	66.7 (66.7)	0.277
Symptom scale**^Ω^	M (SD)	25.9 (21.6)	17.7 (14.0)		20.3 (18.2)	19.9 (15.1)		19.5 (16.8)	20.9 (17.3)		18.6 (17.6)	21.9 (16.2)		17.3 (14.5)	21.6 (18.2)		18.0 (16.9)	21.8 (16.9)		17.9 (14.2)	32.6 (24.2)		19.7 (15.7)	21.7 (21.6)	
	Me (IR)	26.5 (28.9)	14.3 (17.3)	0.060	14.9 (21.9)	17.8 (23.8)	0.899	15.5 (23.2)	17.8 (19.1)	0.673	12.6 (23.8)	19.0 (24.4)	0.337	13.9 (22.6)	17.2 (22.1)	0.229	11.9 (19.5)	19.0 (26.4)	0.262	13.8 (19.8)	28.6 (27.3)	0.052	16.7 (19.7)	16.7 (34.3)	0.636
Side effects of systemic therapyβ	M (SD)	23.0 (21.8)	16.8 (15.5)		20.2 (18.9)	16.3 (15.7)		18.2 (17.5)	19.3 (18.2)		15.7 (17.5)	21.9 (17.6)		16.4 (15.2)	19.9 (19.1)		17.0 (17.1)	19.9 (18.2)		16.4 (14.8)	31.1 (24.6)		18.3 (17.1)	19.8 (20.5)	
	Me (IR)	16.7 (23.8)	9.5 (23.8)	0.256	14.3 (23.8)	9.5 (23.8)	0.385	16.7 (23.8)	9.5 (23.8)	0.655	9.5 (19.0)	19.0 (28.6)	0.025	11.9 (19.0)	16.7 (23.8)	0.522	14.3 (19.0)	14.3 (23.8)	0.358	9.5 (21.4)	28.6 (38.1)	0.045	14.3 (23.8)	11.9 (26.2)	0.986
Concerns about hair lossβ	M (SD)	48.5 (50.2)	28.8 (40.2)		40.6 (47.1)	23.3 (35.3)		22.2 (37.9)	51.1 (46.9)		37.2 (45.5)	33.3 (43.9)		23.1 (39.4)	43.9 (47.2)		21.4 (36.1)	45.6 (47.4)		21.8 (35.2)	100 (0.0)		33.3 (43.0)	41.7 (49.6)	
	Me (IR)	33.3 (100)	0.0 (66.7)	0.272	0.0 (100)	0.0 (33.3)	0.398	0.0 (33.3)	66.7 (100)	0.082	0.0 (100)	0.0 (83.3)	0.811	0.0 (33.3)	33.3 (100)	0.191	0.0 (33.3)	33.3 (100)	0.183	0.0 (33.3)	100 (0.0)	0.001	0.0 (66.7)	16.7 (100)	0.659
Arm-related symptomsβ	M (SD)	31.8 (31.2)	23.8 (27.8)		22.8 (27.3)	31.2 (30.9)		23.7 (28.4)	29.8 (29.9)		24.7 (31.0)	27.9 (26.7)		21.3 (27.1)	28.8 (30.1)		22.5 (25.8)	29.2 (31.2)		23.3 (26.4)	43.6 (34.9)		26.3 (28.9)	25.6 (29.9)	
	Me (IR)	27.8 (55.6)	11.1 (33.3)	0.190	11.1 (33.3)	22.2 (44.4)	0.114	11.1 (33.3)	22.2 (44.4)	0.191	11.1 (44.4)	22.2 (44.4)	0.206	11.1 (33.3)	16.7 (55.6)	0.187	11.1 (33.3)	16.7 (55.6)	0.285	11.1 (33.3)	33.3 (44.4)	0.029	11.1 (44.4)	16.7 (33.3)	0.906
Breast-related symptomsβ	M (SD)	25.6 (25.5)	13.9 (16.7)		17.1 (20.6)	17.9 (20.2)		19.0 (21.8)	15.0 (17.9)		18.2 (22.3)	16.5 (18.0)		15.7 (17.9)	17.9 (21.6)		16.7 (21.3)	18.0 (19.7)		16.4 (18.7)	21.8 (30.1)		16.6 (18.7)	20.8 (16.1)	
	Me (IR)	25.0 (33.3)	8.3 (25.0)	0.025	8.3 (25.0)	8.3 (25.0)	0.708	8.3 (33.3)	8.3 (25.0)	0.478	8.3 (25.0)	8.3 (33.3)	0.919	8.3 (29.2)	8.3 (25.0)	0.789	8.3 (25.0)	8.3 (29.2)	0.595	8.3 (29.2)	8.3 (25.0)	0.857	8.3 (25.0)	8.3 (25.0)	0.623

α The differences are justified by the lack of information; β p-value of Mann–Whitney U Test; ^Ω^ p-value of Student’s *t*-test; * higher scores indicate better quality of life.; ** Higher scores indicate worse quality of life.

M: mean; SD: standard deviation; Me: median; IR: interquartile range.

White women (*versus* black women) scored worse on the future perspectives scale of the EORTC QLQ-BR23. Women with eight or more years of education (*versus* those with fewer years of education) scored better on the physical and role function scales of the EORTC QLQ-C30 and the sexual functioning scale of the EORTC QLQ-BR23. Who lived with a partner (*versus* those without one) scored better on the functional, physical function, role function, cognitive function, insomnia, appetite, and financial difficulties scales of the EORTC QLQ-C30 and the sexual functioning and side effects of systemic therapy scales of the EORTC QLQ-BR23.

Women with a paying job (*versus* those without one) scored better on the social function and financial difficulties scales of the EORTC QLQ-C30 and the sexual functioning scale of the EORTC QLQ-BR23. Women who received care from private healthcare (*versus* those who received it from public healthcare) scored better on the role function scale of the EORTC QLQ-C30. Women with a per capita income equal to or higher than half of the minimum wage (*versus* those with a lower per capita income) scored better on the global health, functional, physical function, role function, emotional function, social function, symptom, fatigue, diarrhea, and financial difficulties scales of the EORTC QLQ-C30 and the side effects of systemic therapy, concerns about hair loss, and arm-related symptoms scales of the EORTC QLQ-BR23. Women living in the municipality where they availed oncology services (*versus* those living in other municipalities) scored better on the global health scale of the EORTC QLQ-C30.

Table 4 presents the results of determining differences in respondents’ scores on the EORTC QLQ-C30 and EORTC QLQ-BR23 based on behavioral factors. Women who engaged in physical activity (*versus* those who did not) scored better on the functional, physical function, cognitive function, and fatigue scales of the EORTC QLQ-C30 and the side effects of systemic therapy scale of the EORTC QLQ-BR23. Women who consumed tobacco (*versus* those who did not) scored worse on the dyspnea scale of the EORTC QLQ-C30 and the side effects of systemic therapy scale of the QLQ-BR23. Religiosity was found to have a mixed impact on respondents’ QOL, as women with high scores on the religiosity scale (*versus* those with low scores) scored worse on the body image scale of the EORTC QLQ-BR23 but better on the breast-related symptoms scale of the EORTC QLQ-BR23. In contrast, women with high scores on the social support questionnaire (*versus* those with low scores) scored better on the functional scale of the EORTC QLQ-C30 and the functional, arm-related symptoms, and breast-related symptoms scales of the EORTC QLQ-BR23.

**Table 4:** Quality of life of the women who participated in the study on the scales of the QLQ-C30 and QLQ-BR23 questionnaires according to behavioral characteristics

		Self-reported eating habits			Level of physical activity^∞^			Tobacco use			Alcohol consumption			Religiosity^µ^			Social support^¥^		
					
		Good or very good	Fair, poor, or very poor	p value	Active or inactive	Sedentary	p value	Ex-smoker or never smoked	Smoker	p value	<4 drinks on one occasion	≥4 drinks on one occasion	p value	≥8 points (mean)	<8 points (mean)	p value	≥45 points (mean)	<45 points (mean)	p value
EORTC QLQ-C30, n (%)α		68 (67.3)	33 (32.7)		77 (76.2)	24 (23.8)		92 (91.1)	9 (8.9)		84 (83.2)	17 (16.8)		36 (35.6)	65 (64.4)		46 (47.4)	51 (52.6)	
Global health scale*β	M (SD)	75.6 (18.6)	71.5 (21.5)		75.9 (18.2)	67.7 (23.1)		75.1 (19.5)	65.7 (20.2)		75.2 (20.1)	69.6 (16.6)		70.6 (19.9)	76.3 (19.3)		76.8 (17.9)	73.9 (20.4)	
	Me (IR)	83.3 (33.3)	75.0 (33.3)	0.397	83.3 (33.3)	66.7 (33.3)	0.202	79.2 (33.3)	58.3 (33.3)	0.142	83.3 (33.3)	66.7 (25.0)	0.196	66.7 (25.0)	83.3 (33.3)	0.189	83.3 (33.3)	75.0 (33.3)	0.558
Functional scale*^Ω^	M (SD)	75.9 (19.8)	76.1 (18.4)		78.2 (18.5)	68.9 (20.3)		76.6 (19.1)	69.9 (21.5)		76.2 (19.8)	75.2 (17.1)		77.9 (16.9)	74.9 (20.5)		77.7 (21.3)	74.9 (17.1)	
	Me (IR)	80.0 (23.3)	80.0 (24.4)	0.972	80.0 (22.2)	70.0 (31.1)	0.038	80.0 (24.4)	73.3 (15.6)	0.388	80.0 (25.6)	75.6 (11.1)	0.846	77.8 (24.4)	80.0 (24.4)	0.462	82.2 (24.4)	75.6 (28.9)	0.479
Physical functionβ	M (SD)	79.7 (19.7)	84.2 (14.6)		83.5 (17.6)	73.9 (18.8)		81.0 (18.5)	82.9 (17.0)		80.9 (18.9)	82.7 (14.5)		83.5 (16.6)	79.9 (19.1)		80.3 (19.9)	82.1 (16.8)	
	Me (IR)	86.7 (26.7)	86.7 (20.0)	0.393	86.7 (13.3)	73.3 (33.3)	0.022	86.7 (23.3)	86.7 (13.3)	0.852	86.7 (26.7)	86.7 (13.3)	0.919	86.7 (13.3)	86.7 (26.7)	0.395	86.7 (26.7)	86.7 (20.0)	0.838
Role functionβ	M (SD)	79.9 (27.8)	76.3 (30.0)		80.1 (29.5)	74.3 (25.0)		80.4 (27.1)	61.1 (37.3)		80.6 (26.9)	69.6 (34.9)		78.7 (30.5)	78.7 (27.6)		81.2 (29.1)	75.8 (28.3)	
	Me (IR)	100 (33.3)	100 (33.3)	0.631	100 (33.3)	66.7 (41.7)	0.171	100 (33.3)	66.7 (67.7)	0.101	100 (33.3)	83.3 (50.0)	0.216	100 (33.3)	100 (33.3)	0.785	100 (33.3)	83.3 (50.0)	0.227
Emotional functionβ	M (SD)	67.4 (30.3)	61.4 (33.8)		67.9 (30.4)	57.6 (34.1)		66.7 (30.9)	52.8 (35.8)		65.7 (32.1)	64.2 (28.8)		68.7 (28.4)	63.6 (33.1)		68.3 (33.9)	63.6 (29.3)	
	Me (IR)	75.0 (45.8)	66.7 (58.3)	0.422	75.0 (41.7)	58.3 (53.3)	0.228	75.0 (50.0)	66.7 (33.3)	0.186	75.0 (50.0)	66.7 (50.0)	0.638	75.0 (41.7)	75.0 (58.3)	0.555	75.0 (58.3)	75.0 (41.7)	0.184
Cognitive functionβ	M (SD)	65.4 (32.4)	70.7 (28.3)		71.9 (28.5)	52.1 (34.5)		67.6 (31.9)	62.9 (21.7)		66.5 (32.4)	70.6 (23.9)		67.6 (31.1)	66.9 (31.2)		76.8 (28.2)	59.1 (32.0)	
	Me (IR)	66.7 (50.0)	83.3 (50.0)	0.525	83.3 (50.0)	50.0 (58.3)	0.011	83.3 (50.0)	66.7 (16.7)	0.349	83.3 (50.0)	66.7 (33.3)	0.915	83.3 (41.7)	83.3 (50.0)	0.951	83.3 (33.3)	66.7 (50.0)	0.003
Social functionβ	M (SD)	90.2 (22.3)	90.4 (19.9)		90.3 (21.2)	90.3 (23.0)		90.6 (21.9)	87.0 (18.2)		90.7 (21.5)	88.2 (21.9)		91.7 (18.0)	89.5 (23.3)		87.3 (24.4)	94.4 (15.5)	
	Me (IR)	100 (8.3)	100 (0.0)	0.985	100 (0.0)	100 (8.3)	0.992	100 (0.0)	100 (16.7)	0.202	100 (0.0)	100 (16.7)	0.593	100 (8.3)	100 (0.0)	0.940	100 (16.7)	100 (0.0)	0.053
Symptom scale**^Ω^	M (SD)	19.7 (16.7)	19.3 (17.5)		18.1 (17.2)	24.8 (15.1)		19.2 (16.0)	24.8 (25.1)		18.6 (16.5)	24.9 (18.5)		19.0 (16.7)	20.0 (17.1)		18.9 (18.7)	20.5 (15.9)	
	Me (IR)	17.9 (25.6)	15.4 (2301)	0.868	12.8 (20.5)	26.9 (23.1)	0.090	15.4 (24.4)	17.9 (17.9)	0.345	15.4 (23.1)	23.1 (17.9)	0.165	14.1 (25.6)	17.9 (20.5)	0.773	14.1 (17.9)	17.9 (25.6)	0.656
Fatigueβ	M (SD)	29.1 (29.2)	25.9 (26.3)		24.8 (27.3)	38.4 (29.1)		27.3 (27.8)	35.8 (33.2)		26.2 (27.6)	37.2 (30.4)		26.8 (26.1)	28.7 (29.5)		27.5 (28.5)	28.3 (28.5)	
	Me (IR)	22.2 (50.0)	22.2 (44.4)	0.691	11.1 (33.3)	38.9 (55.6)	0.036	22.2 (44.4)	22.2 (44.4)	0.335	11.1 (44.4)	22.2 (33.3)	0.087	22.2 (38.9)	22.2 (44.4)	0.965	22.2 (22.2)	22.2 (44.4)	0.991
Nauseaβ	M (SD)	7.6 (16.6)	4.0 (8.4)		6.9 (15.4)	4.9 (11.5)		6.3 (14.4)	7.4 (15.9)		5.6 (13.3)	10.8 (19.5)		8.3 (16.7)	5.4 (13.2)		6.5 (15.1)	6.9 (14.6)	
	Me (IR)	0.0 (0.0)	0.0 (0.0)	0.653	0.0 (0.0)	0.0 (0.0)	0.727	0.0 (0.0)	0.0 (0.0)	0.987	0.0 (0.0)	0.0 (16.7)	0.375	0.0 (16.7)	0.0 (0.0)	0.347	0.0 (0.0)	0.0 (16.7)	0.712
Painβ	M (SD)	33.1 (33.3)	31.3 (36.7)		29.2 (32.9)	43.1 (37.1)		33.7 (34.8)	20.4 (27.4)		31.7 (35.2)	36.3 (29.6)		26.4 (29.9)	35.9 (36.2)		29.3 (32.6)	36.3 (36.0)	
	Me (IR)	16.7 (58.3)	16.7 (66.7)	0.599	16.7 (50.0)	25.0 (50.0)	0.067	16.7 (66.7)	16.7 (16.7)	0.275	16.7 (58.3)	33.3 (50.0)	0.334	16.7 (33.3)	16.7 (66.7)	0.295	16.7 (50.0)	33.3 (66.7)	0.359
Dyspneaβ	M (SD)	6.4 (15.5)	4.0 (11.0)		6.9 (15.6)	1.4 (6.8)		4.7 (13.6)	14.8 (17.6)		5.2 (14.2)	7.8 (14.6)		7.4 (14.0)	4.6 (14.3)		6.5 (15.1)	4.6 (13.4)	
	Me (IR)	0.0 (0.0)	0.0 (0.0)	0.561	0.0 (0.0)	0.0 (0.0)	0.091	0.0 (0.0)	0.0 (33.3)	0.012	0.0 (0.0)	0.0 (0.0)	0.299	0.0 (0.0)	0.0 (0.0)	0.147	0.0 (0.0)	0.0 (0.0)	0.441
Insomniaβ	M (SD)	29.9 (38.7)	30.3 (38.5)		27.3 (36.2)	38.9 (44.7)		28.6 (37.8)	44.4 (44.1)		30.9 (40.0)	25.5 (30.1)		30.6 (35.9)	29.7 (40.0)		31.2 (39.4)	28.8 (38.9)	
	Me (IR)	0.0 (66.7)	0.0 (33.3)	0.842	0.0 (33.3)	16.7 (100)	0.315	0.0 (66.7)	33.3 (100)	0.201	0.0 (66.7)	33.3 (33.3)	0.960	33.3 (50.0)	0.0 (66.7)	0.607	0.0 (66.7)	0.0 (66.7)	0.773
Loss of appetiteβ	M (SD)	5.9 (19.0)	18.2 (35.4)		9.1 (25.1)	12.5 (29.2)		8.3 (24.0)	25.9 (40.1)		9.5 (25.6)	11.8 (28.7)		12.0 (29.9)	8.7 (23.8)		10.1 (27.9)	9.1 (24.1)	
	Me (IR)	0.0 (0.0)	0.0 (0.0)	0.051	0.0 (0.0)	0.0 (0.0)	0.384	0.0 (0.0)	0.0 (66.7)	0.086	0.0 (0.0)	0.0 (0.0)	0.725	0.0 (0.0)	0.0 (0.0)	0.663	0.0 (0.0)	0.0 (0.0)	0.795
Constipationβ	M (SD)	23.0 (36.1)	28.3 (40.1)		20.3 (33.8)	38.9 (44.7)		25.0 (36.9)	22.2 (44.1)		23.8 (36.8)	29.4 (40.6)		29.6 (41.9)	22.0 (34.5)		20.3 (34.8)	29.4 (39.8)	
	Me (IR)	0.0 (33.3)	0.0 (66.7)	0.587	0.0 (33.3)	16.7 (100)	0.059	0.00 (33.3)	0.0 (0.0)	0.546	0.0 (33.3)	0.0 (66.7)	0.612	0.0 (66.7)	0.0 (33.3	0.499	0.0 (33.3)	0.0 (66.7)	0.199
Diarrheaβ	M (SD)	7.8 (20.9)	2.0 (8.1)		5.6 (18.3)	6.9 (16.9)		5.07 (15.6)	14.8 (33.8)		4.4 (14.4)	13.7 (29.0)		5.6 (16.9)	6.1 (18.5)		7.2 (22.1)	4.6 (13.4)	
	Me (IR)	0.0 (0.0)	0.0 (0.0)	0.188	0.0 (0.0)	0.0 (0.0)	0.447	0.00 (0.0)	0.0 (0.0)	0.289	0.0 (0.0)	0.0 (0.0)	0.094	0.0 (0.0)	0.0 (0.0)	0.869	0.0 (0.0)	0.0 (0.0)	0.995
Financial difficultiesβ	M (SD)	17.2 (30.7)	19.2 (34.4)		19.5 (33.0)	12.5 (27.5)		15.94 (30.2)	37.0 (42.3)		15.5 (29.9)	29.4 (38.9)		12.0 (26.6)	21.0 (34.1)		15.9 (31.2)	18.9 (32.8)	
	Me (IR)	0.0 (33.3)	0.0 (33.3)	0.875	0.0 (33.3)	0.0 (0.0)	0.294	0.00 (33.3)	33.3 (66.7)	0.061	0.0 (33.3)	0.0 (33.3)	0.084	0.0 (0.0)	0.0 (33.3)	0.188	0.0 (33.3)	0.0 (33.3)	0.581
EORTC QLQ BR-23, n (%)α																			
Functional scale*^Ω^	M (SD)	63.1 (16.4)	59.4 (18.8)		62.2 (18.4)	60.8 (12.7)		62.8 (17.1)	52.8 (16.5)		63.3 (15.5)	55.1 (19.4)		59.4 (16.6)	63.3 (17.5)		65.7 (16.1)	58.8 (17.6)	
	Me (IR)	66.7 (20.8)	61.9 (21.4)	0.319	66.7 (25.0)	66.7 (13.1)	0.733	66.7 (20.8)	57.1 (24.4)	0.096	66.7 (20.8)	62.5 (16.7)	0.076	61.9 (19.9)	66.7 (22.6)	0.286	68.7 (26.8)	61.9 (21.4)	0.049
Body imageβ	M (SD)	83.7 (24.6)	80.3 (25.5)		81.6 (26.8)	85.8 (17.3)		83.3 (23.6)	75.0 (34.1)		84.7 (23.4)	72.1 (29.4)		79.2 (22.9)	84.5 (25.8)		87.3 (20.5)	78.8 (27.6)	
	Me (IR)	91.7 (25.0)	91.7 (33.3)	0.360	91.7 (25.0)	91.7 (25.0)	0.867	91.7 (25.0)	91.7 (33.3)	0.480	91.7 (25.0)	83.3 (50.0)	0.059	83.3 (33.3)	100 (25.0)	0.025	100 (25.0)	91.7 (25.0)	0.058
Future perspectivesβ	M (SD)	52.9 (42.0)	45.4 (44.7)		52.4 (42.4)	44.4 (44.7)		51.8 (42.9)	37.0 (42.3)		53.2 (43.0)	37.2 (40.6)		49.1 (40.2)	51.3 (44.7)		52.9 (42.5)	47.7 (43.3)	
	Me (IR)	66.7 (100)	33.3 (100)	0.425	66.7 (100)	33.3 (100)	0.472	66.7 (100)	33.3 (66.7)	0.342	66.7 (100)	33.3 (66.7)	0.143	50.0 (100)	66.7 (100)	0.786	66.7 (100)	33.3 (100)	0.599
Sexual functioningβ	M (SD)	32.1 (28.3)	25.8 (34.9)		32.7 (30.6)	21.5 (29.3)		30.6 (30.3)	24.1 (34.5)		28.8 (31.5)	36.3 (25.2)		29.6 (30.9)	30.3 (30.6)		34.4 (30.7)	26.1 (29.1)	
	Me (IR)	33.3 (50.0)	0.0 (50.0)	0.142	33.3 (50.0)	0.0 (33.3)	0.093	33.3 (50.0)	0.0 (33.3)	0.403	16.7 (50.0)	33.3 (33.3)	0.201	33.3 (50.0)	33.3 (50.0)	0.880	33.3 (50.0)	16.7 (50.0)	0.174
Sexual satisfactionβ	M (SD)	50.0 (38.9)	61.5 (38.1)		52.4 (38.5)	53.3 (42.2)		54.7 (39.3)	33.3 (29.8)		54.3 (39.9)	46.1 (34.8)		48.5 (42.1)	54.9 (37.0)		52.1 (38.7)	55.1 (38.8)	
	Me (IR)	66.7 (100)	66.7 (66.7)	0.339	66.7 (66.7)	66.7 (100)	0.942	66.7 (66.7)	33.3 (66.7)	0.186	66.7 (100)	33.3 (33.3)	0.472	33.3 (100)	66.7 (66.7)	0.576	66.7 (83.3)	66.7 (66.8)	0.765
Symptom scale**^Ω^	M (SD)	20.3 (17.7)	19.6 (15.6)		19.5 (17.9)	22.2 (13.5)		19.2 (16.0)	29.4 (23.6)		19.7 (16.9)	22.3 (17.7)		16.7 (15.6)	21.9 (17.5)		17.7 (18.8)	21.8 (15.3)	
	Me (IR)	16.1 (25.2)	17.8 (20.0)	0.848	14.3 (23.8)	21.8 (20.5)	0.489	16.1 (21.9)	26.2 (16.7)	0.085	16.7 (20.9)	20.0 (21.4)	0.557	9.5 (18.6)	19.0 (23.8)	0.137	13.8 (19.0)	19.0 (24.4)	0.274
Side effects of systemic therapyβ	M (SD)	18.6 (18.1)	18.6 (17.2)		17.2 (18.1)	23.2 (15.8)		17.3 (16.6)	32.3 (23.4)		18.1 (17.4)	21.0 (19.6)		19.0 (16.8)	18.4 (18.3)		17.2 (19.5)	19.4 (16.6)	
	Me (IR)	14.3 (23.8)	14.3 (23.8)	0.858	9.5 (23.8)	21.4 (28.6)	0.044	9.5 (21.4)	28.6 (23.8)	0.029	9.5 (23.8)	19.0 (23.8)	0.684	14.3 (16.7)	14.3 (23.8)	0.572	9.5 (19.0)	19.0 (23.8)	0.278
Concerns about hair lossβ	M (SD)	31.9 (44.4)	43.3 (44.6)		31.9 (42.0)	43.3 (49.8)		29.9 (41.2)	75.0 (50.0)		35.9 (44.1)	33.3 (47.1)		24.4 (40.7)	44.4 (45.7)		47.1 (48.7)	24.4 (36.7)	
	Me (IR)	0.0 (100)	33.3 (100)	0.398	0.0 (66.7)	16.7 (100)	0.544	0.0 (66.7)	100 (50.0)	0.082	0.0 (100)	0.0 (100)	0.903	0.0 (33.3)	33.3 (100)	0.200	33.3 (100)	0.0 (33.3)	0.220
Arm-related symptomsβ	M (SD)	25.8 (28.3)	26.9 (30.8)		26.5 (29.9)	25.0 (26.3)		25.8 (28.6)	29.6 (342)		25.4 (29.4)	30.1 (27.1)		20.4 (28.5)	29.4 (28.9)		21.0 (28.3)	30.7 (29.4)	
	Me (IR)	11.1 (38.9)	11.1 (44.4)	0.959	11.1 (44.4)	22.2 (33.3)	0.873	11.1 (44.4)	22.2 (33.3)	0.793	11.1 (38.9)	22.2 (33.3)	0.270	11.1 (33.3)	22.2 (55.6)	0.086	11.1 (33.3)	22.2 (44.4)	0.037
Breast-related symptomsβ	M (SD)	18.7 (22.5)	14.6 (14.9)		17.7 (21.9)	16.3 (14.4)		17.1 (20.3)	20.4 (21.7)		17.2 (20.0)	18.6 (22.5)		9.5 (12.6)	21.8 (22.5)		14.3 (21.6)	19.1 (18.0)	
	Me (IR)	8.3 (33.3)	8.3 (25.0)	0.846	8.3 (25.0)	12.5 (20.8)	0.605	8.3 (25.0)	25.0 (25.0)	0.645	8.3 (25.0)	8.3 (41.7)	0.866	4.2 (16.7)	16.7 (33.3)	0.004	4.2 (25.0)	8.3 (33.3)	0.040

α The differences are justified by the lack of information; β p-value of Mann-Whitney U Test; ^Ω^ p-value of Student’s *t*-test; ^∞^ Assessed using the International Physical Activity Questionnaire – Short Form; Active: Engaged in physical activity for at least 150 minutes in the last week; Inactive: Engaged in physical activity for less than 150 minutes in the last week; Sedentary: Did not engage in physical activity for at least ten consecutive minutes in the last week; ^µ^Assessed using the Duke University Religiosity Index, in which the total sample obtained an average of 8 points; ^¥^Assessed by the Social Support Questionnaire – Short Form, in which the total sample obtained an average of 45 points; * higher scores indicate better quality of life; ** higher scores indicate worse quality of life.

M: mean; SD: standard deviation; Me: median; IR: interquartile range.

Table 5 presents the results of determining differences in respondents’ scores on the EORTC QLQ-C30 and EORTC QLQ-BR23 based on clinical factors. Women with normal weight (*versus* those who were overweight) scored better on the pain scale of the EORTC QLQ-C30 and the arm-related symptoms scale of the QLQ-BR23. Women with at least one comorbidity (*versus* those without a comorbidity) scored worse on the physical function and insomnia scales of the EORTC QLQ-C30. Women who underwent lumpectomy (*versus* those who underwent mastectomy) scored better on the role function and appetite scales of the EORTC QLQ-C30 and the sexual functioning and concerns about hair loss scales of the EORTC QLQ-BR23.

**Table 5:** Quality of life of the women who participated in the study on the scales of the QLQ-C30 and QLQ-BR23 questionnaires according to clinical characteristics

		Body mass index^Þ^			Presence of comorbiditiesφ			Surgical intervention			Stage		
			
		Normal weight	Overweight	p value	Yes	No	p value	Lumpectomy	Mastectomy with or without reconstruction	p value	Initial (0, I, or II)	Advanced (III)	p value
EORTC QLQ-C30, n (%)^1^		33 (35.1)	61 (64.9)		67 (66.3)	34 (33.7)		91 (91.0)	9 (9.0)		79 (78.2)	22 (21.8)	
Global health scale*β	M (SD)	76.5 (18.6)	73.4 (20.7)		71.6 (19.5)	79.4 (19.0)		74.7 (19.8)	70.4 (19.6)		74.4 (19.6)	73.9 (20.3)	
	Me (IR)	83.3 (25.0)	75.0 (33.3)	0.550	75.0 (25.0)	83.3 (33.3)	0.059	75.0 (33.3)	75.0 (33.3)	0.464	75.0 (33.3)	75.0 (33.3)	0.943
Functional scale*^Ω^	M (SD)	78.4 (18.7)	74.5 (20.3)		73.7 (19.6)	80.5 (18.1)		76.4 (19.7)	71.6 (16.3)		76.3 (20.6)	74.8 (13.9)	
	Me (IR)	84.4 (26.7)	77.8 (24.4)	0.358	75.6 (28.9)	86.7 (24.4)	0.098	80.0 (26.7)	71.1 (17.8)	0.480	82.2 (26.7)	75.6 (15.6)	0.754
Physical functionβ	M (SD)	84.8 (13.5)	78.9 (20.7)		78.3 (19.7)	86.9 (13.6)		81.2 (19.0)	82.2 (8.8)		81.6 (18.0)	79.7 (19.5)	
	Me (IR)	86.7 (13.3)	86.7 (33.3)	0.344	80.0 (33.3)	86.7 (20.0)	0.036	86.7 (26.7)	80.0 (6.7)	0.506	86.7 (20.0)	80.0 (26.7)	0.626
Role functionβ	M (SD)	79.3 (29.8)	78.4 (28.3)		78.1 (26.9)	79.9 (31.7)		80.2 (28.7)	66.7 (23.6)		80.6 (28.5)	71.9 (27.9)	
	Me (IR)	100 (33.3)	100 (33.3)	0.738	83.3 (33.3)	100 (33.3)	0.380	100 (33.3)	66.7 (16.7)	0.036	100 (33.3)	66.7 (50.0)	0.093
Emotional functionβ	M (SD)	67.9 (30.7)	63.9 (32.8)		62.6 (33.1)	71.1 (27.5)		65.9 (31.1)	57.4 (36.4)		65.5 (32.9)	65.1 (26.3)	
	Me (IR)	83.3 (41.7)	75.0 (50.0)	0.555	75.0 (50.0)	75.0 (25.0)	0.309	75.0 (50.0)	66.7 (66.7)	0.485	75.0 (50.0)	66.7 (50.0)	0.562
Cognitive functionβ	M (SD)	68.2 (29.6)	65.6 (33.0)		63.7 (31.1)	74.0 (30.2)		67.4 (30.8)	61.1 (34.4)		66.7 (32.0)	68.9 (27.8)	
	Me (IR)	83.3 (50.0)	83.3 (50.0)	0.836	66.7 (50.0)	83.3 (33.3)	0.080	83.3 (50.0)	66.7 (50.0)	0.580	83.3 (50.0)	66.7 (50.0)	0.899
Social functionβ	M (SD)	92.9 (19.1)	89.6 (22.4)		90.3 (21.7)	90.2 (21.4)		90.5 (21.9)	88.9 (18.6)		90.1 (23.2)	90.9 (14.3)	
	Me (IR)	100 (0.0)	100 (16.7)	0.378	100 (0.0)	100 (16.7)	0.575	100 (0.0)	100 (16.7)	0.525	100 (0.0)	100 (16.7)	0.278
Symptom scale**^Ω^	M (SD)	17.8 (17.3)	20.6 (17.3)		21.5 (16.4)	15.9 (17.6)		19.2 (16.9)	24.8 (17.9)		20.2 (18.3)	17.8 (10.3)	
	Me (IR)	12.8 (20.5)	17.9 (23.1)	0.449	17.9 (23.1)	11.5 (20.5)	0.119	15.4 (25.6)	23.1 (15.4)	0.353	12.8 (25.6)	19.2 (12.8)	0.438
Fatigueβ	M (SD)	25.2 (30.7)	29.7 (28.0)		30.5 (28.4)	23.2 (27.7)		27.6 (28.2)	34.6 (30.6)		28.9 (29.4)	24.7 (23.9)	
	Me (IR)	11.1 (44.4)	22.2 (33.3)	0.253	22.2 (44.4)	11.1 (33.3)	0.165	22.2 (44.4)	33.3 (44.4)	0.466	22.2 (44.4)	16.7 (22.2)	0.792
Nauseaβ	M (SD)	8.6 (15.7)	5.2 (14.1)		5.7 (13.1)	7.8 (17.0)		6.8 (15.1)	3.7 (7.3)		7.4 (15.9)	3.0 (6.6)	
	Me (IR)	0.0 (16.7)	0.0 (0.0)	0.169	0.0 (0.0)	0.0 (0.0)	0.791	0.0 (0.0)	0.0 (0.0)	0.850	0.0 (0.0)	0.0 (0.0)	0.451
Painβ	M (SD)	20.7 (25.3)	39.6 (37.7)		36.6 (35.4)	24.5 (30.8)		31.3 (34.7)	40.7 (30.2)		31.0 (35.9)	37.9 (27.3)	
	Me (IR)	16.7 (33.3)	16.7 (66.7)	0.023	16.7 (66.7)	16.7 (50.0)	0.069	16.7 (66.7)	33.3 (33.3)	0.222	16.7 (66.7)	41.7 (33.3)	0.154
Dyspneaβ	M (SD)	3.0 (9.7)	6.6 (15.9)		5.9 (14.1)	4.9 (14.5)		5.9 (14.6)	3.7 (11.1)		5.5 (14.5)	6.1 (13.2)	
	Me (IR)	0.0 (0.0)	0.0 (0.0)	0.311	0.0 (0.0)	0.0 (0.0)	0.564	0.0 (0.0)	0.0 (0.0)	0.719	0.0 (0.0)	0.0 (0.0)	0.659
Insomniaβ	M (SD)	31.3 (39.0)	29.5 (39.9)		37.3 (41.6)	15.7 (26.2)		29.3 (37.8)	40.7 (46.5)		33.3 (39.9)	18.2 (30.4)	
	Me (IR)	0.0 (66.7)	0.0 (66.7)	0.669	33.3 (66.7)	0.0 (33.3)	0.015	0.0 (66.7)	33.3 (100)	0.439	0.0 (66.7)	0.0 (33.3)	0.106
Loss of appetiteβ	M (SD)	16.2 (32.4)	6.6 (21.8)		10.4 (27.3)	8.8 (23.6)		6.6 (20.6)	44.4 (47.1)		11.4 (28.2)	4.5 (15.6)	
	Me (IR)	0.0 (0.0)	0.0 (0.0)	0.063	0.0 (0.0)	0.0 (0.0)	0.930	0.0 (0.0)	33.3 (100)	0.001	0.00 (0.0)	0.0 (0.0)	0.359
Constipationβ	M (SD)	19.2 (32.3)	26.2 (38.5)		25.9 (40.1)	22.5 (31.5)		25.3 (36.9)	22.2 (44.1)		24.9 (37.9)	24.2 (35.9)	
	Me (IR)	0.0 (33.3)	0.0 (33.3)	0.508	0.0 (33.3)	0.0 (33.3)	0.887	0.0 (33.3)	0.0 (0.0)	0.534	0.0 (33.3)	0.0 (33.3)	0.829
Diarrheaβ	M (SD)	4.0 (11.0)	7.1 (21.2)		7.5 (19.9)	2.9 (12.6)		6.6 (18.7)	0.0 (0.0)		6.7 (18.8)	3.0 (14.2)	
	Me (IR)	0.0 (0.0)	0.0 (0.0)	0.960	0.0 (0.0)	0.0 (0.0)	0.189	0.0 (0.0)	0.0 (0.0)	0.249	0.0 (0.0)	0.0 (0.0)	0.250
Financial difficultiesβ	M (SD)	23.2 (35.8)	14.2 (30.7)		17.4 (32.5)	18.6 (30.9)		17.9 (31.9)	18.5 (33.8)		16.9 (32.8)	21.2 (28.3)	
	Me (IR)	0.0 (33.3)	0.0 (0.0)	0.132	0.0 (33.3)	0.0 (33.3)	0.529	0.0 (33.3)	0.0 (33.3)	0.876	0.0 (33.3)	0.0 (33.3)	0.156
EORTC QLQ BR-23, n (%)^1^													
Functional scale*^Ω^	M (SD)	64.1 (15.9)	61.6 (17.5)		62.2 (15.6)	61.2 (20.2)		62.0 (17.6)	59.6 (14.5)		61.8 (17.3)	62.1 (17.2)	
	Me (IR)	66.7 (20.8)	62.5 (21.4)	0.494	66.7 (19.0)	66.7 (25.0)	0.776	66.7 (22.6)	66.7 (23.2)	0.690	66.7 (19.0)	62.5 (21.4)	0.958
Body imageβ	M (SD)	84.6 (18.4)	82.9 (27.5)		84.2 (23.0)	79.4 (28.1)		82.0 (25.7)	86.1 (15.6)		82.6 (24.6)	82.6 (26.1)	
	Me (IR)	91.7 (25.0)	91.7 (25.0)	0.453	91.7 (25.0)	91.7 (33.3)	0.534	91.7 (25.0)	91.7 (33.3)	0.995	91.7 (25.0)	95.8 (25.0)	0.773
Future perspectivesβ	M (SD)	45.4 (43.1)	53.0 (43.2)		51.7 (43.5)	48.0 (41.9)		49.4 (42.6)	55.6 (47.1)		48.5 (42.6)	57.6 (43.9)	
	Me (IR)	33.3 (100)	66.7 (100)	0.422	66.7 (100)	50.0 (100)	0.654	66.7 (100)	66.7 (100)	0.668	33.3 (100)	66.7 (100)	0.425
Sexual functioningβ	M (SD)	34.8 (31.3)	28.4 (30.2)		28.4 (31.8)	33.3 (28.1)		32.4 (30.6)	9.3 (22.2)		30.4 (30.0)	28.8 (30.0)	
	Me (IR)	33.3 (50.0)	33.3 (50.0)	0.305	16.7 (50.0)	33.3 (50.0)	0.309	33.3 (50.0)	0.0 (0.0)	0.021	33.3 (50.0)	16.7 (66.7)	0.745
Sexual satisfactionβ	M (SD)	63.5 (33.2)	44.4 (40.6)		47.7 (41.2)	60.6 (33.5)		52.0 (39.3)	66.7 (0.0)		54.4 (37.7)	43.3 (44.6)	
	Me (IR)	66.7 (66.7)	33.3 (83.3)	0.079	66.7 (100)	66.7 (66.7)	0.233	66.7 (100)	66.7 (0.0)	0.665	66.7 (66.7)	33.3 (100)	0.439
Symptom scale**^Ω^	M (SD)	18.9 (17.5)	20.7 (17.2)		20.9 (16.9)	18.6 (17.2)		19.8 (16.9)	24.9 (16.6)		20.4 (17.9)	19.2 (13.3)	
	Me (IR)	11.1 (26.7)	17.8 (17.3)	0.636	17.8 (17.8)	13.1 (24.1)	0.529	16.7 (24.3)	21.4 (8.9)	0.399	16.7 (21.9)	17.3 (23.8)	0.787
Side effects of systemic therapyβ	M (SD)	21.4 (19.7)	17.6 (17.1)		20.3 (17.7)	15.3 (17.5)		18.0 (17.3)	25.9 (21.9)		19.8 (18.4)	14.5 (14.7)	
	Me (IR)	19.0 (23.8)	9.5 (23.8)	0.436	19.0 (23.8)	9.5 (14.3)	0.083	14.3 (23.8)	23.8 (23.8)	0.246	14.3 (23.8)	7.1 (28.6)	0.201
Concerns about hair lossβ	M (SD)	35.7 (46.2)	37.0 (44.1)		36.0 (45.0)	33.3 (43.6)		29.9 (43.0)	75.0 (31.9)		34.6 (43.8)	38.9 (49.1)	
	Me (IR)	0.0 (100)	16.7 (100)	0.834	0.0 (100)	16.7 (66.7)	0.963	0.0 (66.7)	83.3 (50.0)	0.034	0.0 (100)	16.7 (100)	0.796
Arm-related symptomsβ	M (SD)	18.8 (28.1)	29.3 (29.6)		27.9 (29.4)	22.9 (28.9)		25.6 (27.8)	34.6 (40.6)		24.9 (29.1)	30.8 (28.7)	
	Me (IR)	11.1 (22.2)	22.2 (55.6)	0.045	11.1 (44.4)	5.6 (33.3)	0.209	11.1 (44.4)	11.1 (44.4)	0.596	11.1 (33.3)	22.2 (55.6)	0.303
Breast-related symptomsβ	M (SD)	13.6 (15.9)	18.8 (22.4)		15.5 (18.0)	21.1 (24.1)		18.2 (20.9)	11.1 (12.5)		17.2 (19.5)	18.2 (23.5)	
	Me (IR)	8.3 (25.0)	8.3 (25.0)	0.339	8.3 (25.0)	8.3 (41.7)	0.368	8.3 (33.3)	8.3 (16.7)	0.417	8.3 (25.00)	8.3 (33.3)	0.716

α The differences are justified by the lack of information; β p-value of Mann-Whitney U Test; ^Ω^ p-value of Student’s *t*-test; ^Þ^Body mass index ≥25 kg/m^2^ was considered overweight; ^φ^ If there was a record of comorbidities such as hypertension, diabetes, dyslipidemia, and depression in the medical record, comorbidities were considered to be present; * higher scores indicate better quality of life; ** higher scores indicate worse quality of life.

M: Mean; SD: standard deviation; Me: Median; IR: interquartile range.

## DISCUSSION

Scores on the EORTC QLQ-C30 and EORTC QLQ-BR23 were similar to those reported in a Brazilian study involving 172 women, most of whom (77.6%) had completed adjuvant treatment for breast cancer.^(17)^

In this study, the global health, functional, and symptom scales of both questionnaires exhibited better QOL scores than the scores reported in studies that evaluated women undergoing chemotherapy or radiotherapy.^(18-20)^

Adjuvant treatment for breast cancer can result in physical, social, and functional changes that affect women’s QOL. Nevertheless, these negative effects tend to gradually decline after the completion of systemic treatment.^(21)^

Our respondents had better scores on the functional scales of social function, physical function, and body image as well as the symptom scales of diarrhea, dyspnea, and nausea. These results indicate their ability to recover their social activities and physical well-being after breast cancer treatment, along with the remission of symptoms related to systemic treatment.

However, the respondents scored lower on the functional scales of emotional function, cognitive function, sexual, and future perspectives. These findings suggest the presence of long-term effects of therapy and concerns about the future, which highlights the need to adopt a broader and more continuous approach by a multi- and interdisciplinary health team. Regarding the symptom scales, the worst scores were associated with pain and insomnia, which are nonspecific symptoms that might be influenced by the patient’s lifestyle rather than being directly linked to breast cancer.

Overall, our results indicate promising improvements in various aspects of patients’ lives after they receive breast cancer treatment. However, they also highlight the importance of addressing the emotional, cognitive, and sexual well-being of these women in the follow-ups.

Regarding the factors related to the post-treatment QOL of women with breast cancer, women aged 50 or more had better scores on the emotional function scale of the EORTC QLQ-C30 and the body image, future perspectives, and breast-related symptoms scales of the EORTC QLQ-BR23 but worse scores on the sexual functioning scale of the EORTC QLQ-BR23. This finding is similar to that of a literature review that found that older women are more mentally prepared to deal with treatment.^(22)^ However, the impact of age on the QOL of patients with breast cancer remains a matter of debate, with some studies indicating that older women may have worse overall QOL.^(23)^

Aging can decrease functional capabilities and QOL, but older women may have already undergone such changes because of other conditions. Thus, the decline in their QOL from breast cancer treatment may not be as severe as that in younger women. Furthermore, young individuals are subjected to society-defined beauty standards, but this subjection is less evident for older individuals.^(24)^

Menopause causes hormonal changes that can be exacerbated by breast cancer treatment.^(25)^ This may explain why older women had lower scores for sexual functioning. A study conducted in Curitiba involving 48 patients with breast cancer obtained similar results.^(26)^

White women had worse scores on the future perspectives scale of the EORTC QLQ-BR23. Race is an indirect indicator of socioeconomic status, access to health services, and information about diseases. Therefore, white women may have more information about the severity and symptoms of breast cancer, causing greater concerns about the future.^(27)^

Women with eight or more years of education had better scores on the physical and role function scales of the EORTC QLQ-C30 and the sexual functioning scale of the EORTC QLQ-BR23. A Polish study involving 324 women with breast cancer also found that women with higher educational levels have better QOL. Higher education levels were found to be associated with a better understanding of health guidelines, the ability to identify changes caused by treatment, and the tendency to seek more health services.^(28)^ Thus, women with higher educational levels may have the opportunity to access therapies and treatments that improve their functional abilities and sexual function. Additionally, education is related to increased health awareness, improvements in self-care, and the ability to cope with the side effects of treatment.^(27,29)^

Women who lived with a partner scored better on the functional, physical function, role function, cognitive function, insomnia, loss of appetite, and financial difficulties scales of the EORTC QLQ-C30 and the sexual function and side effects of systemic therapy scales of the EORTC QLQ-BR23. This result is consistent with the findings of a Polish study that found that married women have higher QOL due to greater family support in coping with the disease.^(28)^

Women who had a paying job had better scores on the social function and financial difficulties scales of the EORTC QLQ-C30 and the sexual functioning scale of the EORTC QLQ-BR23. This result aligns with the findings of a study conducted in Barretos, São Paulo, involving 304 women with breast cancer. It found that women who return to work after treatment have higher QOL. Having a job can provide social contact and financial stability, contributing to a higher QOL. This may explain, at least in part, the higher scores on the social function, financial difficulties, and sexual functioning scales. However, economically active women generally have better health, allowing them to work.^(30)^

Women receiving care from the private healthcare system scored better on the role function scale of the EORTC QLQ-C30. This finding aligns with that of a study conducted in Curitiba. It found that patients with breast cancer receiving private care have better QOL. In private services, access regulation does not follow the principle of hierarchy. Thus, access to specialized care can be facilitated, which can lead to less aggressive treatment and access to specific therapies, resulting in an improved QOL.^(31)^

Women with a per capita income equal to or higher than half of the minimum wage scored better on the global health, functional, physical function, role function, emotional function, social function, symptoms, fatigue, diarrhea, and financial difficulties scales of the EORTC QLQ-C30. They also scored higher on the side effects of systemic therapy, concerns about hair loss, and arm-related symptoms scales of the EORTC QLQ-BR23. This result is consistent with the findings of a study conducted in Vitória, Espírito Santo.^(32)^ A higher income can provide greater access to healthcare and disease information as well as treatments that alleviate side effects, leading to better QOL.^(28)^

Women living in the municipality where they availed oncology services scored better on the global health scale of the EORTC QLQ-C30. Similar results were found in a Polish study involving 250 women with breast cancer. The study found that being close to well-equipped healthcare centers grants women with breast cancer access to medical specialists, medical tests, and health information, leading to better QOL.^(29)^

Physically active women scored better on the functional, physical function, cognitive function, and fatigue scales of the EORTC QLQ-C30 and the side effects of systemic therapy scale of the EORTC QLQ-BR23. An intervention study involving women diagnosed with breast cancer and aromatase inhibitors found improvements in the QOL domains of the group that engaged in physical activity. Physical activity can improve several aspects of QOL, such as functional performance, strength, aerobic capacity, and sleep quality, and reduce adverse effects.^(33)^

Women who consumed tobacco scored worse on the dyspnea scale of the EORTC QLQ-C30 and the side effects of systemic therapy scale of the EORTC QLQ-BR23. A study in Poland involving 250 women with breast cancer also found that tobacco use results in lower QOL. Tobacco consumption harms the pulmonary and circulatory systems, thereby negatively affecting one’s QOL.^(29)^

Women with high scores on the religiosity scale scored worse on the body image scale of the EORTC QLQ-BR23 but better on the breast-related symptoms scale of the EORTC QLQ-BR23. A study conducted in Porto Alegre evaluated 108 women with breast cancer and found a correlation between QOL and spirituality. Spirituality is an effective strategy for reducing suffering and can provide comfort, faith, peace, and a sense of purpose during challenging times.^(19)^

Women with high scores on the social support questionnaire scored better on the cognitive function scale of the EORTC QLQ-C30 and the functional, arm-related symptoms, and breast-related symptoms scales of the EORTC QLQ-BR23. A Chinese study involving 98 women with breast cancer found that women with more social support have better QOL. Social support helps individuals cope with adverse situations, such as breast cancer, and positively affects both physical and mental health, especially under stressful conditions.^(34)^

Women with normal weight scored better on the pain scale of the EORTC QLQ-C30 and the arm-related symptoms scale of the EORTC QLQ-BR23. This result is consistent with the findings of a Polish study involving 250 women with breast cancer. The study found that obesity is a well-established risk factor for the development, progression, and recurrence of breast cancer, can negatively influence treatment effectiveness, and cause complications.^(29)^ Body weight is often linked to one’s lifestyle and significantly affects QOL.

Women with at least one comorbidity scored worse on the physical function and insomnia scales of the EORTC QLQ-C30. A study involving 114 American women with breast cancer found worse QOL in patients with comorbidities.^(35)^ The presence of comorbidities can worsen functional limitations and symptoms associated with the disease, thereby affecting various aspects of QOL.^(29)^

Women who underwent lumpectomy scored better on the role and loss of appetite scales of the EORTC QLQ-C30 and the sexual functioning and concerns about hair loss scales of the EORTC QLQ-BR23. A study conducted in Florianópolis, Brazil, involving 172 women with breast cancer found that women who underwent radical surgery had lower QOL scores than those who underwent lumpectomy.^(17)^ Breasts have a cultural and social significance in women’s experience of sexuality, and any threat to its integrity can cause feelings of inferiority, rejection, and loss of self-esteem. Surgical intervention and the side effects of systemic treatments can lead to psycho-emotional challenges for women with breast cancer, impacting their body image and sexuality.^(21)^

Owing to improvements in therapies, the QOL of patients undergoing breast cancer treatment has improved in recent years. However, aspects such as emotional function, body image, sexual function, and concerns about future perspectives require attention from healthcare professionals.

In Brazil, primary healthcare, which is considered the gateway to the healthcare network, plays a key role in monitoring patients with breast cancer, especially more vulnerable patients. This monitoring can be improved by establishing matrix support and shared care between primary care and referral centers for cancer care. However, not all Brazilian municipalities have full coverage of primary care services. Moreover, the workload of primary care teams and the large number of incomplete teams, especially teams of community health agents, pose challenges to providing this care.

Due to the complex nature of the construct of QOL, multidisciplinary teams and intersectoral partnerships must be developed to provide effective breast cancer treatment and overcome access barriers and health inequalities in Brazil.

Despite the limitations stemming from the subjectivity of QOL and its measurement, we used validated instruments to minimize bias. Although the sample size was not too large, no differences were observed between the women included in the study (n=101) and those eligible for the study (n=129) in terms of sociodemographic factors such as age, education, race, marital status, comorbidities, stage of illness, and type of care. Thus, the risk of bias due to unequal loss of participants was reduced.

Additionally, measuring QOL at least three years after diagnosis resulted in greater homogeneity among the study population. It also allowed us to analyze long-term effects in women who had not relapsed, minimizing the impact of the initial discovery and acceptance of the disease and the often more aggressive therapy administered during that phase.

## CONCLUSION

Higher post-treatment quality of life of women with breast cancer is associated with being Black, being 50 years old or older, having eight or more years of education, having a partner, having a paying job, receiving care from the private healthcare system, having a per capita income equal to or more than half of the minimum wage, living in the municipality where the healthcare service is located, engaging in physical activity, not consuming tobacco, being highly religious, having more social support, not being overweight, having no comorbidities, and undergoing lumpectomy.

These findings suggest that sociodemographic, clinical, and lifestyle factors influence the quality of life of women with breast cancer, even after a few years of diagnosis. Interventions aimed at promoting health and reducing inequalities in access to healthcare can mitigate cancer-related symptoms and enhance the quality of life of survivors of breast cancer, even after the end of chemotherapy or radiotherapy.

The results of this study broaden our understanding of sociodemographic, behavioral, and clinical factors that influence post-treatment quality of life of Brazilian women diagnosed with breast cancer. Longitudinal studies should be conducted to investigate the correlations reported in this study. They should also include variables such as environmental conditions, multidisciplinary support, and mental health factors.
